# Role of macrophage autophagy in postoperative pain and inflammation in mice

**DOI:** 10.1186/s12974-023-02795-w

**Published:** 2023-05-02

**Authors:** Kazuha Mitsui, Sohei Hishiyama, Aakanksha Jain, Yumi Kotoda, Masako Abe, Takashi Matsukawa, Masakazu Kotoda

**Affiliations:** 1 Department of Anesthesiology, Faculty of Medicine, University of Yamanashi, 1110 Shimokato, Chuo, Yamanashi 409-3898 Japan; 2 F.M. Kirby Neurobiology Center, Boston Children’s Hospital, and Department of Neurobiology, Harvard Medical School, 3 Blackfan Circle, Boston, MA 02115 USA; 3 Department of Ophthalmology, Faculty of Medicine, University of Yamanashi, 1110 Shimokato, Chuo, Yamanashi 409-3898 Japan

**Keywords:** Autophagy, Inflammation, Macrophage, Pain, Surgery

## Abstract

**Background:**

Postoperative pain and inflammation are significant complications following surgery. Strategies that aim to prevent excessive inflammation without hampering natural wound-healing are required for the management of postoperative pain and inflammation. However, the knowledge of the mechanisms and target pathways involved in these processes is lacking. Recent studies have revealed that autophagy in macrophages sequesters pro-inflammatory mediators, and it is therefore being recognized as a crucial process involved in regulating inflammation. In this study, we tested the hypothesis that autophagy in macrophages plays protective roles against postoperative pain and inflammation and investigated the underlying mechanisms.

**Methods:**

Postoperative pain was induced by plantar incision under isoflurane anesthesia in mice lacking macrophage autophagy (Atg5flox/flox LysMCre +) and their control littermates (Atg5flox/flox). Mechanical and thermal pain sensitivity, changes in weight distribution, spontaneous locomotor activity, tissue inflammation, and body weight were assessed at baseline and 1, 3, and 7 days after surgery. Monocyte/macrophage infiltration at the surgical site and inflammatory mediator expression levels were evaluated.

**Results:**

Atg5flox/flox LysMCre + mice compared with the control mice exhibited lower mechanical and thermal pain thresholds and surgical/non-surgical hindlimb weight-bearing ratios. The augmented neurobehavioral symptoms observed in the Atg5flox/flox LysMCre + mice were associated with more severe paw inflammation, higher pro-inflammatory mediator mRNA expression, and more monocytes/macrophages at the surgical site.

**Conclusion:**

The lack of macrophage autophagy augmented postoperative pain and inflammation, which were accompanied by enhanced pro-inflammatory cytokine secretion and surgical-site monocyte/macrophage infiltration. Macrophage autophagy plays a protective role in postoperative pain and inflammation and can be a novel therapeutic target.

**Supplementary Information:**

The online version contains supplementary material available at 10.1186/s12974-023-02795-w.

## Background

Postoperative pain is one of the most significant post-surgical complications and can delay functional recovery and impair patients’ quality of life. Inflammation plays crucial roles in the pathophysiology of postoperative pain. Once tissue damage occurs from surgical intervention, inflammatory cells proliferate at the surgical site, producing inflammatory mediators and causing tissue inflammation [1, 2]. At the site of inflammation, the so-called inflammatory soup—a wide array of signaling molecules, such as cytokines, chemokines, and neurotransmitters—is generated, which triggers inflammatory pain [3]. Conversely, inflammation also functions as a protective innate immune response that is essential for normal wound-healing and tissue remodeling [1]. Therefore, a simple anti-inflammatory strategy may not be ideal for the treatment of postoperative pain, and often raises concerns regarding adverse effects, such as delayed wound-healing [4]. Non-steroidal anti-inflammatory drugs, local anesthetics, and opioids have been widely used to treat postoperative pain; however, many of these drugs have immunosuppressive effects and can delay tissue recovery [4–9]. Therefore, strategies that aim to prevent excessive inflammation without hampering natural wound-healing, rather than those that simply suppress inflammation, are required for the management of postoperative pain and inflammation. However, the knowledge of the mechanisms and target pathways involved in these processes is lacking.

Autophagy, an intracellular self-degradation system, is an essential biological process that removes unnecessary cytosol, cytoplasmic organelles, and even microbes from cells [10–12]. Recent studies have revealed that autophagy in macrophages sequesters pro-inflammatory mediators, and it is therefore being recognized as a crucial process involved in regulating inflammation in various diseases, including atherosclerosis [13, 14], inflammatory bowel disease [15], uveitis [16], infection [17–19], peripheral neuronal injury [20], and brain ischemia [21]. Considering that macrophages are key cells in the generation and resolution of inflammatory pain [1, 22, 23], autophagy in macrophages can be expected to play pivotal roles in postoperative pain and inflammation as well as their resolution, and is therefore a potential therapeutic target.

This study aimed to investigate the potential involvement and protective roles of macrophage autophagy in postoperative pain and inflammation, and to describe the underlying mechanisms.

## Methods

All experiments were conducted in accordance with the National Institutes of Health, National Academy of Science, and International Association for the Study of Pain guidelines for the care and use of laboratory animals. The experimental protocol was reviewed and approved by the University of Yamanashi Animal Care Committee (Approval code: A2-7).

### Animals

Autophagy-related protein 5 (ATG5) is an essential protein for auto-phagosome formation; hence, ATG5 deficiency hinders autophagy [24]. Atg5flox/flox (Atg5 f/f, B6.129S-Atg5 < tmlMyok >) and LysMCre (B6.129S-B6.129P2-Lysm < tml(cre)Ifo >) mice were provided by the RIKEN BRC through the National BioResource Project of the MEXT/AMED, Japan. Atg5 f/f mice bear alleles in which exon 3 of the Atg5 genes is loxP-flanked [24]. Atg5 f/f mice were bred with LysMCre mice to generate mice with autophagy-deficient macrophages (Atg5f/f LysMCre mice). Atg5 f/f littermates served as the controls. C57BL/6J mice were purchased from Japan SLC (Tokyo, Japan). Animals were housed in groups of 4–5 mice. The room temperature of the animal facility was kept at 23 ± 2 °C with 12 h cycles of light and dark. Animals had a free access to water and standard food. Adult male and female mice (10–12 weeks old) were used in this study.

### Isolation of primary peritoneal macrophages and induction of autophagy

Thioglycolate medium (4%, 2 mL, Sigma-Aldrich Japan, Tokyo, Japan) was injected into the peritoneal cavity to elicit macrophages. Four days after the injection, macrophages were collected via peritoneal lavage using 5 mL of cold phosphate-buffered saline (PBS). The harvested cells were centrifuged, counted, re-suspended, and cultured in RPMI-1640 (Thermo Fisher Scientific KK, Tokyo, Japan) for 6 h; fetal bovine serum was not added to induce autophagy under energy-depletion conditions. Subsequently, the culture plates were rinsed twice with RPMI-1640 to remove non-adherent cells, after which the adherent macrophages were harvested.

### Genotyping (polymerase chain reaction, PCR)

DNA from tail cells and primary peritoneal macrophages was extracted using the DNeasy Blood and Tissue Kit (Qiagen KK; Tokyo, Japan). The presence of Cre recombinase and the conditions of Atg5 alleles were evaluated using PCR. The forward (5′-CCCAGAAATGCCAGATTACG-3′) and reverse (5′-GCATTGCAGACTAGCTAAAGGCAG-3′) primers were used to detect Cre recombinase; the resulting Cre product was 800 bp. Atg5 wild type and floxed alleles were distinguished using Atg5 wild type-specific forward (5′-GAATATGAAGGCACACCCCTGAAATG-3′) and Atg5 flox-specific forward (5′-ACAACGTCGAGCACAGCTGCGCAAGG-3′) primers and Atg5 common reverse primer (5′-GTACTGCATAATGGTTTAACTCTTGC-3′). The resulting Atg5 wild type product was 1160 bp, and the resulting Atg5 floxed product was 700 bp, while the deleted Atg5 fragment was 300 bp.

### Western blot analysis

The primary peritoneal macrophages were lysed in RIPA buffer (R0278, Sigma-Aldrich, Missouri, USA) containing protease inhibitors (P8340, P5726, P0044, Sigma-Aldrich) for 30 min on ice and then centrifuged for 10 min at 12,000 g at 4 °C. The supernatants were diluted in 2 × Laemmli sample buffer (#1610737, BIO-RAD, CA, USA) containing 5% β-mercaptoethanol (25099-30, Kanto Chemical Co., Inc., Tokyo, Japan) and heated for 5 min at 95 °C. Lysates (10 µg) were separated using a 10% SDS–polyacrylamide gel electrophoresis and then transferred to nitrocellulose membranes (#1,704,156, BIO-RAD) according to the manufacturer’s instructions. Membranes were blocked in 5% skim milk (Morinaga, Tokyo, Japan) in Tris-buffered saline–Tween (TBST; 20 mM Tris–HCl, 150 mM NaCl, 0.1% Tween-20) for 2 h at room temperature and then incubated with antibodies against ATG5 (1:500; NB110-53818SS, Novus Biologicals, Colorado, USA), LC3 (1:250;NB100-2331SS, Novus Biologicals, Colorado, USA), or SQSTM1/p62 (1:250; MAB8028, R&D Systems, Minneapolis, USA) in 5% skim milk in TBST at 4 °C overnight. Blots were then rinsed and incubated with the following secondary antibodies: anti-rabbit IgG HRP-conjugated (1:1000; #7074, Cell Signaling Technology, MA, USA), anti-mouse IgG HRP-conjugated (1:1000; #7076, Cell Signaling Technology), and anti-β-actin-HRP-Direct (1:1000; PM053-7, MBL Co., Ltd, Tokyo, Japan). Protein bands were detected using an enhanced chemiluminescence detection kit (GE Healthcare Japan, Tokyo, Japan).

### Autophagy assay (immunofluorescence)

The primary peritoneal macrophages were fixed in cold methanol for 10 min, washed three times with PBS, and then incubated in 1% bovine serum albumin plus 0.3% Triton X-100 (12967-32, NACALAI TESQUE, INC., Kyoto, Japan) in PBS for 2 h to block nonspecific protein binding. Macrophages were then stained with Alexa Fluor 635-conjugated rabbit anti-mouse ionized calcium-binding adapter molecule 1 (Iba1, 1:200; 013-26471, Fujifilm Wako Pure Chemical Corporation, Osaka, Japan) and Alexa Fluor 488-conjugated rabbit anti-mouse LC3 (1: 200; NB600-1384AF488, Novus Biologicals, Colorado, USA) overnight in the dark at room temperature. They were then rinsed three times with PBS, followed by nuclear staining (Hoechst 33342 solution, SQ104, Dojindo Laboratories, Kumamoto, Japan). Finally, the immunofluorescent images were visualized using a confocal microscope (A1R HD25, Nikon, Tokyo, Japan) under 800 × magnification, and the density of LC3 immuno-staining was quantified using ImageJ software (National Institutes of Health, Bethesda, MD, USA). Threshold RGB intensity was set in a blinded fashion and maintained throughout the quantification, and the density was recorded as a percentage value of the area of signals that exhibited fluorescence above the threshold.

### Postoperative pain and inflammation

Postoperative pain and inflammation were induced by plantar incision as previously described [25, 26]. Briefly, general anesthesia was initiated using 3% isoflurane and maintained with 2% isoflurane. A 5-mm longitudinal incision was made on the left paw plantar surface using a No.11 surgical blade. The surgical wound was closed with two single sutures of 5–0 nylon. Mechanical and thermal sensitivity, weight-bearing imbalance between the hind paws, spontaneous locomotor activity, paw thickness, and body weight were assessed at baseline and postoperative days 1, 3, and 7. Another set of mice was euthanized on postoperative day 1 or 3 for histological analysis and to evaluate the mRNA levels of inflammatory mediators using immunofluorescence and real-time PCR, respectively.

### Neurobehavioral analysis

All neurobehavioral experiments were carried out by blinded individuals between 9:00 and 18:00 and at 23 ± 2 °C under normal room light. Prior to the experiments, the animals were acclimatized to the testing environment for 1 h for 2 consecutive days for each neurobehavioral test. The tests were repeated at baseline and 1, 3, and 7 days after surgery.

### Mechanical nociceptive threshold

Mechanical hypersensitivity was assessed using the von Frey test [27]. Animals were placed in a plastic chamber on an elevated floor with a metal grid, and von Frey filaments (bending forces: 0.04, 0.07, 0.16, 0.4, 0.6, 1, 1.4, 2, and 4 g) were gently applied from below for 1 s. Positive responses were defined as flinching, shaking, licking, or retraction of the leg, representing a clear nociceptive perception to the mechanical stimulus [28]. The experiments started with the 0.6-g filament, and the up–down method was used to determine the 50% withdrawal threshold [28, 29].

### Thermal nociceptive threshold

Thermal withdrawal latencies were assessed using Hargreaves method [30]. Briefly, animals were placed in the same plastic chamber used for the von Frey test on an elevated glass platform preheated at 29–30 °C. A radiant heat source (NeoHaroBeam, Toshiba, Tokyo, Japan) was positioned underneath the glass floor such that radiant heat was focused on the plantar surface of the paw. The radiant heat intensity was pre-adjusted such that the withdrawal latencies of the naïve paws were approximately 10 s. Heat stimulation was repeated three times at an interval of at least 3 min for each paw, and the results were averaged to determine the mean withdrawal latency (cutoff time 30 s).

### Weight-bearing imbalance

Weight-bearing distribution between the surgical and non-surgical hindlimbs was assessed using the incapacitance test [31]. Briefly, after habituation to the testing environment, each mouse was gently placed in a plastic cage with an inclined floor with each hindlimb resting on two separate pressure sensors. (HJ-150A, A&D, Tokyo, Japan). When a mouse was in a stable position for at least 3 s, the weight-bearing of the left and right hindlimbs was recorded, and the ratio was calculated. Trials were repeated three times, and mean values were recorded.

### Spontaneous locomotor activity

Spontaneous locomotor activity after surgery was assessed using the open-field test as previously described [32, 33]. Animals were set loose in a 50 × 50 × 50-cm plastic chamber with 4 × 4 grid lines on the floor, making a total of 25 10 × 10 cm squares. The number of lines crossed with all paws in 5 min was recorded.

### Infiltration of monocytes/macrophages (immunofluorescence)

The mouse paws were dissected, and 20 µm-thick frozen sections were prepared using a cryostat (CM 1540S, Leica Biosystems, Tokyo, Japan). The sections were fixed in cold methanol for 10 min, washed three times with PBS, and then incubated in 1% bovine serum albumin plus 0.3% Triton X-100 (NACALAI TESQUE) in PBS for 2 h to block nonspecific protein binding. The sections were then stained with Red fluorochrome 635-conjugated rabbit anti-mouse Iba1 (1:200, Fujifilm Wako Pure Chemical Corporation) overnight in the dark at room temperature, and rinsed three times with PBS, followed by nuclear staining (Hoechst 33342 solution, Dojindo Laboratories). Finally, the section images were visualized using a confocal microscope (A1R HD25, Nikon). Using another set of samples, M1/M2 phenotypic differentiation was evaluated using Alexa Fluor 594-conjugated rabbit anti-mouse CD11c (1:200, 117346, BioLegend, San Diego, CA, USA) and Alexa 488-conjugated rabbit anti-mouse CD206 (1:200, 141709, BioLegend, San Diego, CA, USA). For each animal, four 0.025-mm^2^ areas within the dermis under 800 × magnification were randomly selected by a blinded individual. The number of cells was recorded to evaluate cellular infiltration [34].

### Pro-inflammatory cytokine expression (real-time PCR)

Real-time PCR was used to measure the mRNA levels of interleukin 1β (IL-1β), interleukin 6 (IL-6), tumor necrosis factor-α (TNF-α), interleukin 10 (IL-10), and transforming growth factor beta 1 (TGF-β1). The target mRNAs were extracted from paw skin tissue using the RNeasy Mini Kit (Qiagen KK, Tokyo, Japan). cDNA was prepared using the QuantiTect Reverse Transcription Kit (Qiagen KK). PCR was conducted on the StepOneTM real-time PCR system (Life Technologies, Carlsbad, CA, USA) using the PowerSYBR^®^ Green PCR Master Mix (Thermo Fisher Scientific, Waltham, MA, USA) and corresponding primers. The data were normalized by geometric averaging of two internal control genes (*Gapdh* and *Actb*) from the same sample [35]. Both internal control gene expression levels were consistent among non-surgical and surgical paw samples (SD of less than 1 and coefficient of variation of less than 10). The levels of the inflammatory mediators are reported in terms of the fold increase with the levels on the contralateral side of the Atg5 f/f mice defined as 1.0.

### Statistical analysis

Statistical analysis was conducted using GraphPad Prism 9 (GraphPad Software, San Diego). Mechanical withdrawal threshold, thermal withdrawal latency, weight-bearing ratio, spontaneous activity, paw thickness, and body weight over 7 days were analyzed using repeated measures two-way analysis of variance (ANOVA) followed by the Sidak multiple comparisons test. Two group comparisons were analyzed using the two-tailed Student’s *t* test. The mRNA levels of inflammatory cytokines were analyzed using one-way ANOVA followed by Tukey’s multiple comparison test. Experimental sample size was determined based on previous experiments showing sufficient power (> 80%) to detect significance with 95% confidence. Data are presented as the mean ± SEM. *P* < 0.05 was considered statistically significant.

## Results

### Confirmation of Atg5 gene knockout and autophagy deficiency in macrophages

**Fig. 1:**
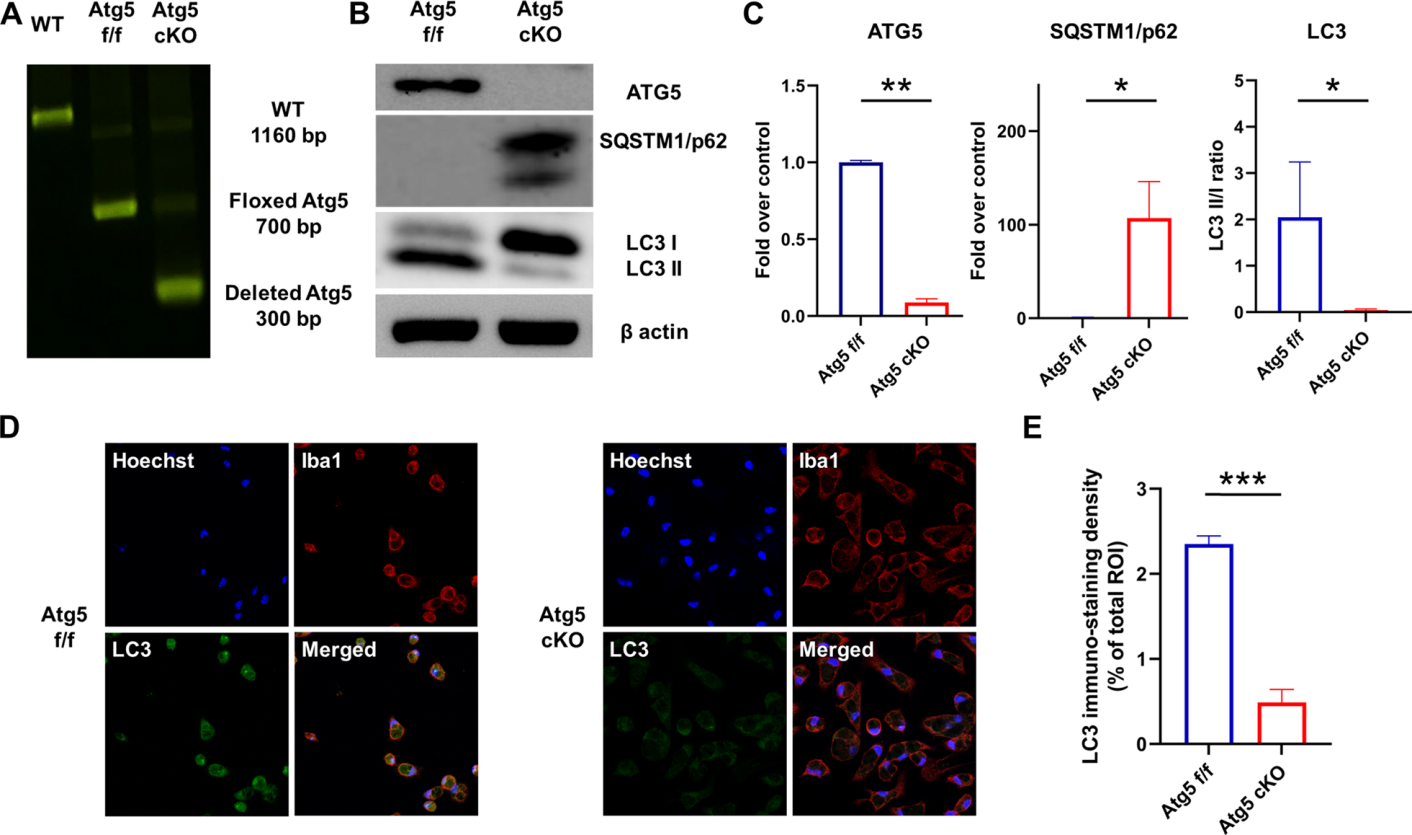
Confirmation of Atg5 gene knockout and autophagy deficiency in macrophages. **A** Polymerase chain reaction analysis of gene in peritoneal macrophages from wild type (WT), Atg5f/f, and Atg5f/fLysMCre (Atg5 cKO) mice. **B** Western blot analysis of ATG5 and SQSTM1/p62 proteins in peritoneal macrophages from Atg5f/f and Atg5f/fLysMCre mice (*n* = 5 each) cultured for 6 h under energy-depletion. **C** Densitometric quantification of western blot of ATG5, SQSTM1/p62, and LC3 proteins (*n* = 3–5 each). **D** Representative immunofluorescence images of macrophages. **E** LC3 immuno-staining density of macrophages (*n* = 3 each). Data are presented as the mean ± SEM. *P < 0.05, **P < 0.01, ***P < 0.001. *cKO* conditional knockout; *WT* wild type

Atg5f/f mice were bred with mice that expressed the Cre recombinase transgene at the lysozyme 2 locus to generate mice that lacked autophagy in macrophages. Representative results of the genotyping of peritoneal macrophages are shown in Fig. 1A. Next, the efficacy of Cre-mediated Atg5 gene deletion and the consequent autophagy deficiency in macrophages were confirmed via western blotting. As shown in Fig. 1B and C, compared with that in control mice, the production of ATG5, a protein essential for autophagy, was significantly impaired in peritoneal macrophages from ATG5 cKO mice (1.0 ± 0.01 vs. 0.09 ± 0.02, *P* < 0.001). Furthermore, Atg5 cKO mice exhibited significant SQSTM1/p62 accumulation (1.0 ± 0.2 vs. 107.0 ± 39.0, *P* < 0.05) and absence of LC3II (LC3 II/I ratio: 2.0 ± 0.7 vs. 0.04 ± 0.01, *P* < 0.05) in macrophages, indicating defunct autophagy in the cells (Fig. 1B and C). Autophagy deficiency in ATG5 cKO mice macrophages was also confirmed using immunofluorescence analysis. As shown in Fig. 1D and E, LC3 immuno-staining density was significantly lower in serum-starved macrophages from ATG5 cKO mice than in those from Atg5 f/f mice (2.35% ± 0.06% vs. 0.49% ± 00.9%, *P* < 0.001).

### Augmented postoperative pain and inflammation in Atg5 cKO mice

**Fig. 2:**
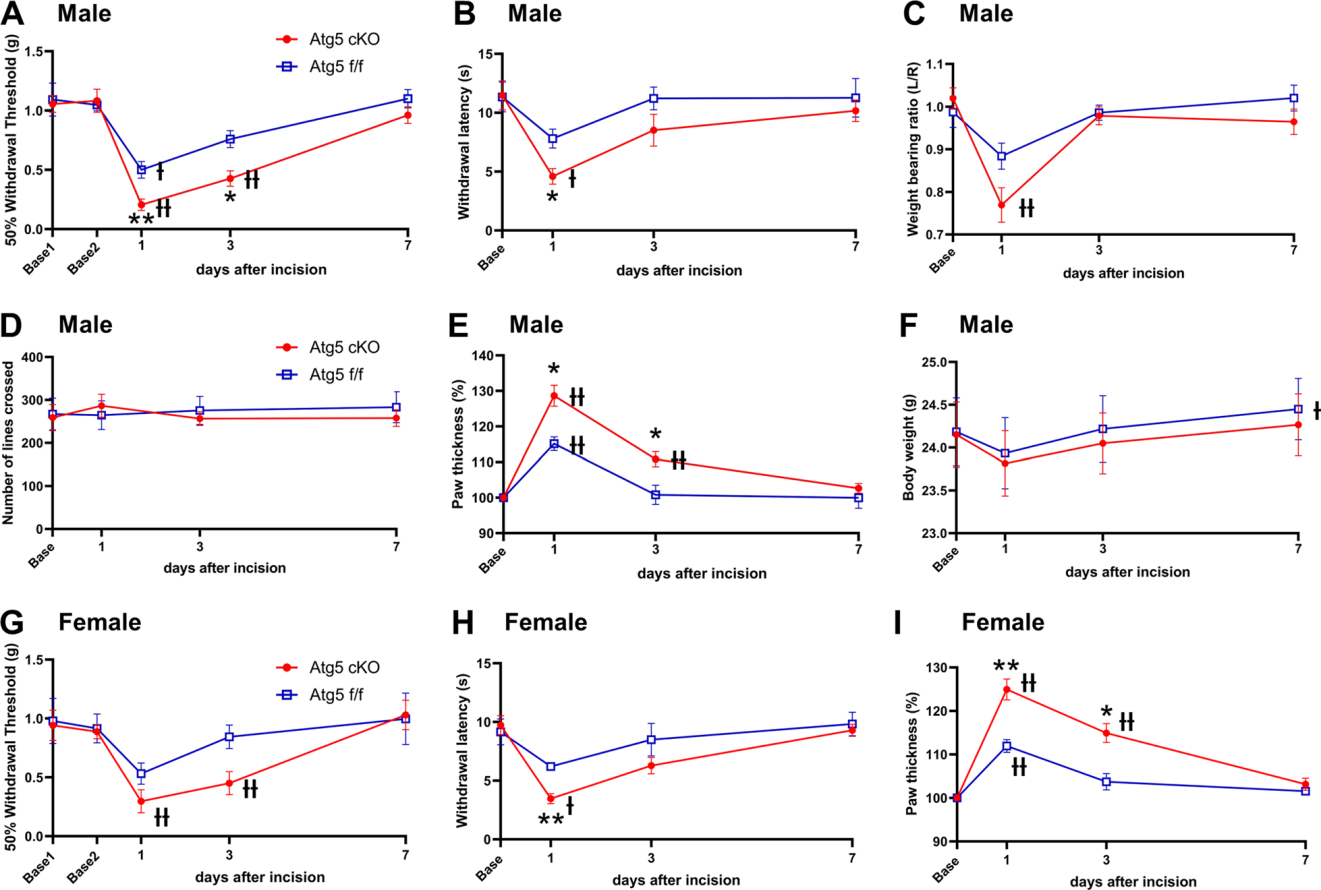
Neurobehavioral analyses. **A**–**F**: male (*n* = 8 each), **G**–**I**: female (*n* = 6 each). **A** Mechanical sensitivity assessed using the von Frey test. **B** Thermal sensitivity assessed using the Hargreaves method. **C** Hind paw weight distribution assessed using the incapacitance test. **D** Spontaneous locomotor activity assessed using the open-field test. **E** Paw edema and inflammation. **F** Body weight. **G** Mechanical sensitivity assessed using the von Frey test. **H** Thermal sensitivity assessed using the Hargreaves method. **I** Paw edema and inflammation. *P < 0.05, **P < 0.01 vs. Atg5 f/f mice. ^†^*P* < 0.05, ^††^*P* < 0.01 vs. baseline within the group. *cKO* conditional knockout

Compared with mice with WT macrophages, Atg5 cKO mice showed more severe mechanical and thermal hypersensitivity and tissue edema after surgery. (Day 1- mechanical threshold: 0.51 ± 0.05 g vs. 0.22 ± 0.05 g, *P* < 0.01; thermal threshold: 7.81 s ± 0.80 s vs. 4.59 s ± 0.66 s, *P* < 0.05; paw thickness: 112.5 ± 2.2% vs. 124.9 ± 3.3%, *P* < 0.05 and Day 3- mechanical threshold: 0.81 g ± 0.06 g vs. 0.49 ± 0.07 g, *P* < 0.05; paw thickness: 100.7 ± 2.0% vs. 109.7 ± 1.8%, *P* < 0.05; male, *n* = 8 each, Fig. 2A–F) Similar results were confirmed in female mice (Fig. 2G–I). Mechanical and thermal sensitivities in the contralateral paws were not affected by the deficient macrophage autophagy (additional study, male, *n* = 6 each, Additional file 1: Figure S1). Although the weight-bearing ratio values were not significantly different between the groups on day 1 (0.88 ± 0.03 vs. 0.77 ± 0.04, *P = *0.159, male), the Atg5 cKO mice exhibited significantly reduced weight-bearing on the surgical side paw (vs. baseline, 1.02 ± 0.02 vs. 0.77 ± 0.04, *P* < 0.01, male). Moreover, although there was no significant difference in body weight between the groups over 7 days, only the control group mice showed a significant increase from the baseline (baseline: 23.78 ± 0.42 g, day 7: 24.05 ± 0.39 g, *P = *0.033) while the Atg5 cKO mice did not (baseline: 23.68 ± 0.43 g, day 7: 23.78 g, *P = *0.358). Postoperative spontaneous locomotor activity was not significantly different between groups (ANOVA, F (1, 14) = 0.298, *P = *0.594).

### Increased monocyte/macrophage infiltration at the surgical site in Atg5 cKO mice

**Fig. 3:**
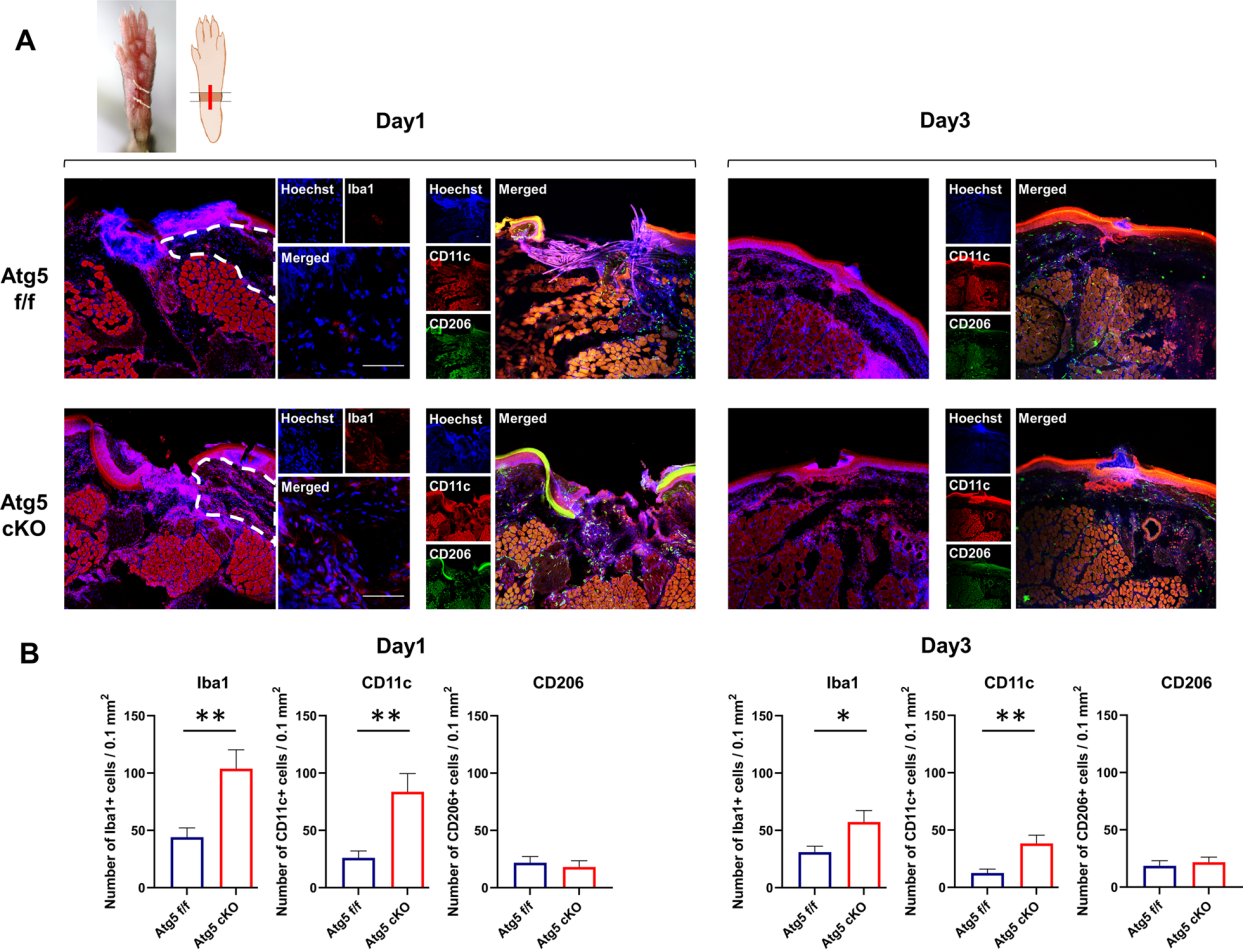
Immunofluorescence analysis. **A** Representative immunofluorescence images of the paw skin tissue, showing the infiltration of ionized calcium-binding adapter molecule 1 (Iba1)-, CD11c-, and CD206-positive cells in the dermis (surrounded by the dotted line). Scale bar: 50 µm. **B** Number of Iba1-, CD11c-, and CD206-positive cells in the dermis (*n* = 6 each). Data are presented as the mean ± SEM. *P < 0.05; **P < 0.01. *cKO* conditional knockout

Figure 3 shows more Iba1-positive and CD11c-positive monocyte/macrophages in the dermis at the surgical site in the Atg5 cKO mice (Day1—Iba1: 44 ± 8 vs. 104 ± 16 cells / 0.1 mm^2^, *n* = 6 each, *P* < 0.01, CD11c: 26 ± 6 vs. 84 ± 16 cells / 0.1mm^2^, *n* = 6 each, *P* < 0.05, Day3—Iba1: 31 ± 5 vs. 57 ± 10 cells / 0.1 mm^2^, *P* < 0.05, CD11c: 13 ± 3 vs. 38 ± 7 cells / 0.1 mm^2^, *P* < 0.01). In contrast, Atg5 cKO and Atg5 f/f mice showed similar numbers of CD206-positive cells in the dermis (Day 1: 22 ± 5 vs. 18 ± 6 cells / 0.1 mm^2^, *P = *0.629, Day 3: 19 ± 5 vs. 22 ± 5 cells / 0.1 mm^2^, *P = *0.633).

### Increased pro-inflammatory cytokine expression in Atg5 cKO mice

**Fig. 4:**
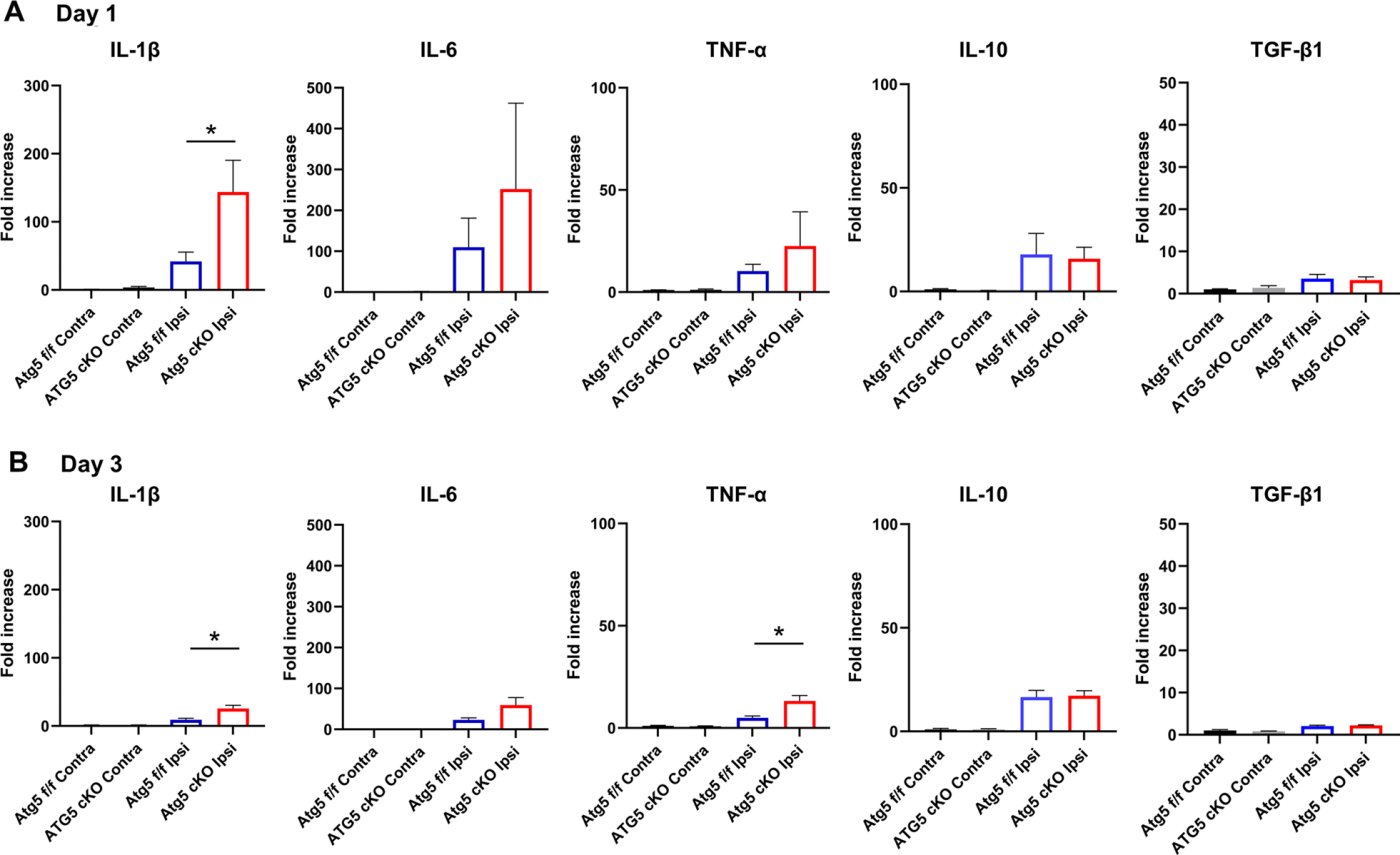
Real-time polymerase chain reaction analysis of the mRNA levels of inflammatory mediators. **A** Day 1 (*n* = 6 each). **B** Day 3 (*n* = 6 each). *P < 0.05. *cKO* conditional knockout, *IL* interleukin, *TGF* transforming growth factor, *TNF* tissue necrosis factor

The mRNA levels of pro-inflammatory cytokines were significantly elevated in the paw skin tissue from the Atg5 cKO mice except for IL-6 (Day 1—IL-1β: 41.8 ± 13.9 vs. 143.8 ± 46.6, Day 3—IL-1β: 9.2 ± 2.2 vs. 25.6 ± 4.8, TNF-α: 5.0 ± 0.9 vs. 13.2 ± 2.7, *P* < 0.05; Fig. 4). In contrast, the mRNA levels of anti-inflammatory mediators (IL-10 and TGF-β1) were not significantly different between groups, demonstrating a shift toward the pro-inflammatory state in the macrophage autophagy-deficient Atg5 cKO mice.

## Discussion

In the present study, we found that a lack of autophagy in macrophages exacerbated postoperative pain and inflammation. Mice lacking macrophage autophagy showed a delay in weight gain after surgery. Moreover, infiltration of monocytes/macrophages at the surgical site and production of pro-inflammatory mediators was enhanced in the mice lacking macrophage autophagy. These observations suggest the significant involvement and protective roles of macrophage autophagy in postoperative pain and inflammation.

Although surgery is a type of treatment, it results in iatrogenic tissue injury. The inflammatory cascade is initiated by inflammatory cytokine secretion from platelets, attracting inflammatory cells, including monocytes/macrophages, to the injured site [36]. Monocytes/macrophages infiltrate, secrete various inflammatory mediators, and engulf pathogens and damaged cells by phagocytosis, leading to tissue inflammation and remodeling [36, 37]. These processes are essential for tissue protection and normal wound-healing; however, they must be controlled in terms of ensuring a balance between pro- and anti-inflammatory elements [38]. Autophagy in macrophages mediates tissue inflammation by continuously removing defunct mitochondria and by regulating inflammasome activation [11, 14, 39]. The tissue-protective effects of macrophage autophagy have been demonstrated in the context of several diseases, as well as infection and ischemia [17, 21]—conditions in which inflammation plays critical roles in the pathogenesis. Our results are consistent with those previous findings, demonstrating that the deficiency of macrophage autophagy results in dysregulation of inflammation and leads to enhanced postoperative inflammation.

In the current study, increased pro-inflammatory mediator gene expression was observed in Atg5 cKO mice. Conversely, the expression of anti-inflammatory mediators was not significantly affected. These observations indicate that the lack of autophagy in monocytes/macrophages caused a shift toward the pro-inflammatory state. Furthermore, the enhancement of pro-inflammatory mediator expression coincided with an increase in the population of M1 monocytes/macrophages in the dermis at the surgical site. Consistent with our findings, earlier studies have reported that autophagy deficiency in myeloid cells induces pro-inflammatory polarization with both enhanced pro-inflammatory cytokine production and infiltration [14, 17, 21, 40], although the mechanism via which macrophage autophagy causes pro-inflammatory polarization remain nebulous. Based on these findings, it would be natural to assume that autophagy deficiency induced augmented pain and inflammation via facilitating pro-inflammatory cytokine production and monocyte/macrophage infiltration. Enhanced production of pro-inflammatory mediators from the autophagy-deficient macrophages presumably attracted more monocytes/macrophages and other inflammatory cells to the injured site, causing a vicious circle of inflammation. These mechanisms would have led to sustained inflammation, thereby contributing to the prolonged neurobehavioral symptoms in Atg5 cKO mice.

Inflammation is a critical component of postoperative pain. Inflammatory mediators activate and sensitize nociceptors, causing spontaneous pain and localized tissue hypersensitivity [22, 41]. Among the various inflammatory cells, macrophages are essential and key mediators of the generation and resolution of inflammatory pain [22, 23]. Infiltrating macrophages sensitize the peripheral nociceptors and facilitate the transition from acute to chronic pain by producing various inflammatory mediators, such as IL-1β and TNF-α [42]. Conversely, macrophages also contribute to the resolution of pain by secreting anti-inflammatory or analgesic effectors [42]. Therefore, it is not surprising that autophagy-related defects in macrophages resulted in the dysregulation of inflammation as well as its resolution, exacerbating and prolonging postoperative pain.

Despite enhanced mechanical pain sensitivity and inflammation, spontaneous locomotor activity was not significantly affected by macrophage autophagy deficiency. This may be explained by the findings of a previous study, which demonstrated that inflammatory pain in one hindlimb is not sufficient to impair the spontaneous activity of young adult mice [43]. However, in our study, weight-bearing imbalance between hindlimbs worsened in the Atg5 cKO mice, indicating that augmented inflammation due to macrophage autophagy deficiency did influence the spontaneous behavior of the mice.

The finding that a lack of macrophage autophagy contributes to postoperative pain and inflammation provides a potential cellular target for the treatment of pain and inflammation associated with surgery. Promotion or enhancement of autophagy in macrophages after surgery may be effective for improving postoperative outcomes [44]. However, autophagy in other cell types could have different roles. Indeed, there are conflicting reports regarding the effects of the indiscriminate modulation of autophagy in various inflammatory diseases, possibly reflecting the diverse roles of autophagy in different cell types [45–48]. Future pharmaceutical development could enable macrophage-specific autophagy enhancement, rendering the potential for a novel strategy for treating postoperative pain and inflammation.

This study has some limitations. Although we focused on autophagy in macrophages, possible interactions and influences of other cell types cannot be ruled out. For example, the increased cytokine mRNA expressions in the paws from Atg5 cKO mice could be an indirect consequence of impaired autophagy in macrophages. Neutrophils also play important roles in the pathogenesis of pain and inflammation after skin incision. However, accumulating evidence has demonstrated that autophagy does not enhance the pro-inflammatory profile of neutrophils or influence morphology or migration ability of neutrophils [49, 50]. Therefore, neutrophil autophagy is less likely to be involved in the augmented pain and inflammation observed in Atg5 cKO mice. In addition, we performed neurobehavioral assays 1, 3, and 7 days after surgery. Data at day 5 would have made the neurobehavioral assay more complete. Finally, we only used relatively young mice to explore a possible role of macrophage autophagy in postoperative pain and inflammation. Future expansive studies using animals at different ages will further add to our findings.

## Conclusions

The lack of macrophage autophagy augmented postoperative pain and inflammation, which were accompanied by enhanced pro-inflammatory cytokine secretion and surgical-site monocyte/macrophage infiltration. Macrophage autophagy plays a protective role in postoperative pain and inflammation and can be a novel therapeutic target.

## Supplementary Information

**Additional file 1**. Neurobehavioral analyses. A and B: male (n = 6 each).** A** Mechanical sensitivity assessed using the von Frey test.** B** Thermal sensitivity assessed using the Hargreaves method.

## Data Availability

The datasets used and/or analyzed during the current study are available from the corresponding author on reasonable request.

## Abbreviations

ANOVA: Analysis of variance
ATG5: Autophagy-related protein 5
cKO: Conditional knockout
IL: Interleukin
PBS: Phosphate-buffered saline
PCR: Polymerase chain reaction
TGF: Transforming growth factor
TNF: Tissue necrosis factor
