# Supplementary figures and images for: Role of macrophage autophagy in postoperative pain and inflammation in mice
Source: J Neuroinflammation. 2023 May 2;20:102. doi: 10.1186/s12974-023-02795-w (PMC10152627; doi:10.1186/s12974-023-02795-w)

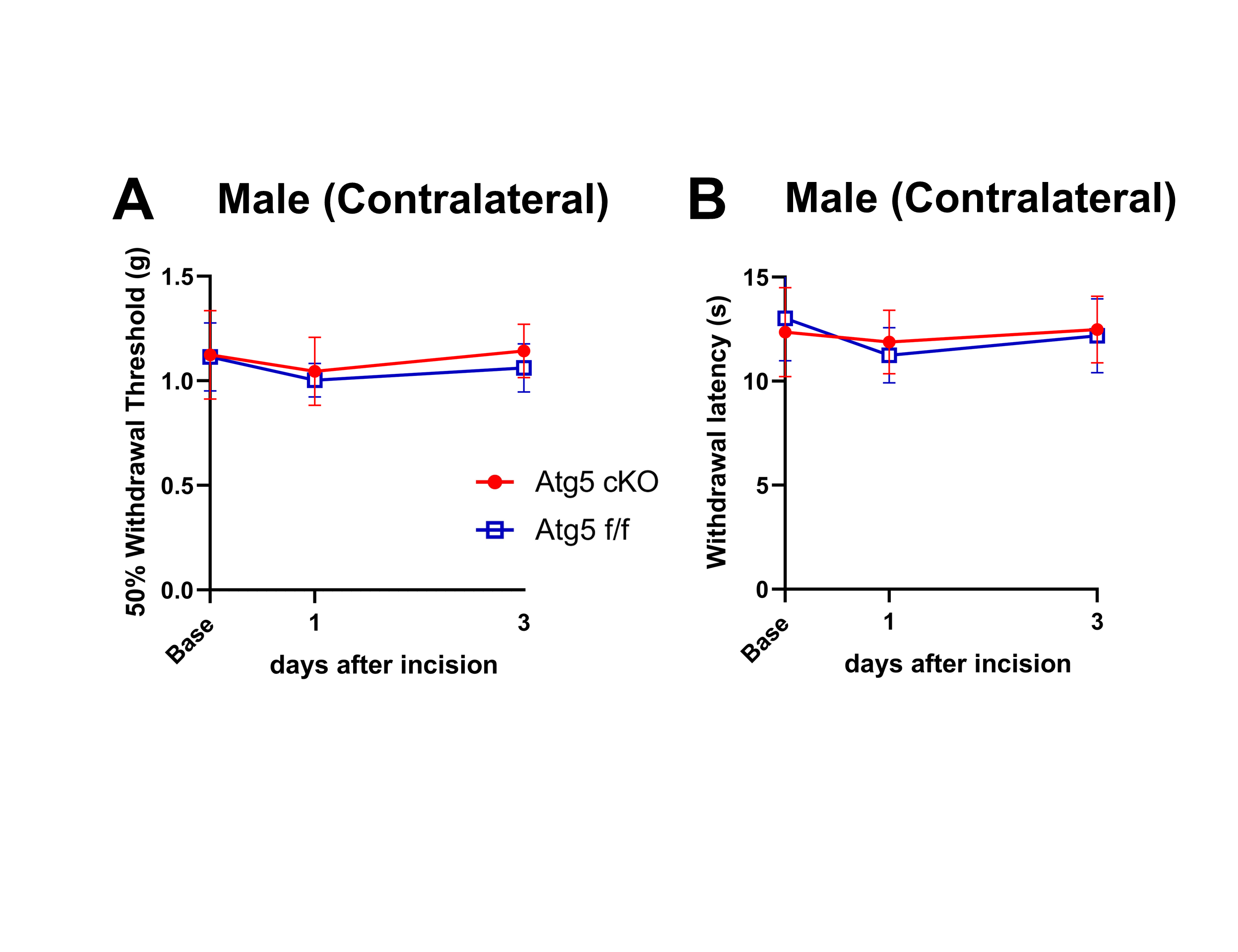

Supplement: Supplementary file 1 — Additional file 1. Neurobehavioral analyses. A and B: male (n = 6 each). A Mechanical sensitivity assessed using the von Frey test. B Thermal sensitivity assessed using the Hargreaves method. [file 12974_2023_2795_MOESM1_ESM.tif]
